# Serum correlation, demographic differentiation, and seasonality of blubber testosterone in common bottlenose dolphins, *Tursiops truncatus,* in Sarasota Bay, FL

**DOI:** 10.1038/s41598-021-88602-z

**Published:** 2021-04-26

**Authors:** Kathryn K. Sherman, Nicole E. Beaulieu-McCoy, Elyse L. Wurster, Randall S. Wells, Cynthia R. Smith, Aaron A. Barleycorn, Jason B. Allen, Nicholas M. Kellar

**Affiliations:** 1 Environmental Assessment Services, LLC, Southwest Fisheries Science Center, National Marine Fisheries Service, National Oceanic and Atmospheric Administration, 8901 La Jolla Shores Drive, La Jolla, CA 92037 USA; 2 Volunteer With Southwest Fisheries Science Center, National Marine Fisheries Service, National Oceanic and Atmospheric Administration, 8901 La Jolla Shores Drive, La Jolla, CA 92037 USA; 3 Chicago Zoological Society’s Sarasota Dolphin Research Program, c/o Mote Marine Laboratory, 1600 Ken Thompson Parkway, Sarasota, FL 34236 USA; 4 National Marine Mammal Foundation, 2240 Shelter Island Drive, Suite 200, San Diego, CA 92106 USA; 5 Southwest Fisheries Science Center, National Marine Fisheries Service, National Oceanic and Atmospheric Administration, 8901 La Jolla Shores Drive, La Jolla, CA 92037 USA

**Keywords:** Endocrinology, Ecology

## Abstract

Blubber and serum testosterone levels were compared among 55 individual common bottlenose dolphins, *Tursiops truncatus*, in Sarasota Bay, FL during 2011–2019. A significant positive relationship between the matrices was found in male testosterone concentrations in 29 paired samples (r^2^ = 0.932). Mature males (n = 17) had 300 times greater mean testosterone concentration in serum than immature males (n = 17). A comparison of blubber samples, including 12 females, 24 immature males, and 19 mature males, revealed significant differences in mean blubber testosterone values among all three demographics. Immature males had greater than 6 times the average blubber testosterone concentration of females and mature males had almost 100 times that of immature males. Estimated testis volume was highly correlated with blubber testosterone concentration and mature males had 60 times greater average testis volume than immature males. We observed seasonal variation in blubber testosterone in mature males, consistent with known reproductive patterns. These data suggest males can be distinguished from females and designated as mature or immature via blubber testosterone concentrations, an observation that validates dart biopsy sampling as a means of obtaining demographic data.

## Introduction

Androgen concentrations, notably testosterone, are commonly used as endocrine indicators of male reproductive maturity in mammals, providing pertinent data on reproduction, development, and seasonal reproductive trends^1^. Testosterone, present in both males and females, is released by the Leydig cells in the testes, and to a lesser extent from the adrenal glands^2–4^. High concentrations of testosterone triggers spermatogenesis and can indicate the onset of sexual maturity or suggest seasonal breeding patterns in mature males^3,5^. Steroid hormones, such as testosterone, first circulate in the blood before incorporating into other matrices such as blubber, hair, and feces. Testosterone levels have been shown to be highly correlated with reproductive success in males^6^. Serum testosterone levels have also been used to investigate breeding seasonality in various cetacean species including the common bottlenose dolphin (*Tursiops truncatus*), beluga whale (*Delphinapterus leucas*), spinner dolphin (*Stenella longirostris*), short-finned pilot whale (*Globicephala macrorhynchus*), and common minke whale (*Balaenoptera acutorostrata*)^6–11^. Significant differences in serum testosterone concentrations with respect to maturity state has been observed in several cetacean species including the Dall’s porpoise (*Phocoenoides dalli*), beluga, bottlenose dolphin, long-finned pilot whale (*Globicephala melas*), and fin whale (*Balaenoptera physalus*)^12–16^. This high variability of testosterone concentrations presents a challenge when examining trends and making assumptions using this biomarker across species, within individuals, and/or across tissue types.

Obtaining serum samples from wild cetaceans is difficult. Blubber, as an alternative to serum, is a more easily accessible matrix to investigate male reproductive seasonality or maturity in free-ranging populations. Remote biopsy sampling has proven to be a relatively simple, safe and effective method of collecting life history and endocrinologically relevant samples from marine mammals^17,18^. There has been an increase in the utility of dart biopsies to obtain valuable biological information on free-ranging cetaceans, including sex, reproductive and stress hormones, environmental contaminants, prey composition by analyzing stable isotopes and fatty acids, age estimation via epigenetics, nutritive condition from percent lipid in blubber, and genetics^19–22^. Biopsy dart sampling has become a useful, multipurpose tool for research, without the risks and expense of capture-release. Dart biopsies can provide baseline data for population evaluations where sex and male maturity state have not been determined. Blubber testosterone concentrations have been used to show maturity and/or seasonality in cetacean species including humpback whales (*Megaptera novaeangliae*)^3^, short-beak common dolphins (*Delphinus delphis*)^1^, and bottlenose dolphins^17,23,24^.

It is important that conclusions made from blubber-derived hormone concentrations are commensurate with those made from blood-derived concentrations as blood has historically been the most widely used matrix for hormone evaluation^25^. Direct comparisons of hormone concentrations in the two matrices are therefore important to validate such determinations. Previous studies have examined paired serum and blubber progesterone and cortisol concentrations^26,27^. Testosterone concentrations have been measured independently in a variety of matrices, including feces, blow, and baleen^26,28,29^. Recently, Galligan et al. (2020) compared plasma and blubber hormone concentrations in bottlenose dolphins but they were unable to obtain quantifiable blubber testosterone concentrations in non-mature male animals using liquid-chromatography tandem-mass spectrometry. Additionally, serum and testis testosterone concentrations have been compared to each other and to morphological measurements of the testes^6,15^. To our knowledge, serum and blubber testosterone concentrations have not been compared in wild cetacean populations.

For dolphins under human care, blood collection and/or morphological measurements of the testes (via ultrasound examination) are part of routine veterinary assessments. For free-ranging populations, testes examination and blood collection are limited to ultrasound examination during capture-release health assessments^30,31^ or necropsy examinations of dead stranded animals.

Sarasota Bay, Florida has a long-term resident community of common bottlenose dolphins with which periodic health assessments have been carried out since the 1980s by the Chicago Zoological Society’s Sarasota Dolphin Research Program (SDRP)^27^. These dolphins have been studied since 1970 making it one of the world’s most thoroughly known populations of small cetaceans, comprised primarily of individuals of known sex, age and genealogy^32^. Capture-release health assessments provide unique opportunities to obtain substantial life-history information on individuals as well as populations^33^. In this study, we compared testosterone concentrations in the serum and blubber of common bottlenose dolphins from Sarasota Bay, FL, in order to understand the relationship between hormone concentrations in the two matrices. We examined the correlation between blubber and serum testosterone concentrations with estimated testis volume to confirm the expected positive relationship. Additionally, we examined if blubber testosterone concentrations can be used to differentiate females, immature males, and mature males. Finally, we compared blubber testosterone values across seasons and demographic groups in order to assess patterns among them.

## Results

**Figure 1:**
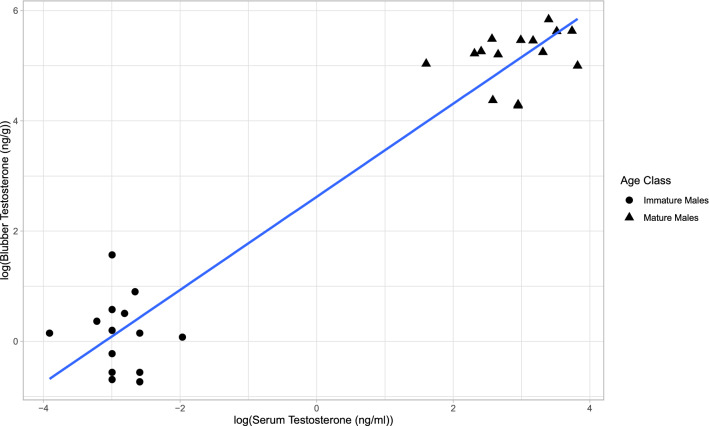
Relationship of blubber testosterone concentration (ng/g lipid mass) to serum testosterone concentration (ng/ml) for mature and immature male *T. truncatus* from Sarasota Bay, FL (r^2^ = 0.932, *p* < 2.2 × 10^–16^, y = 0.845*x + 2.618).

**Figure 2:**
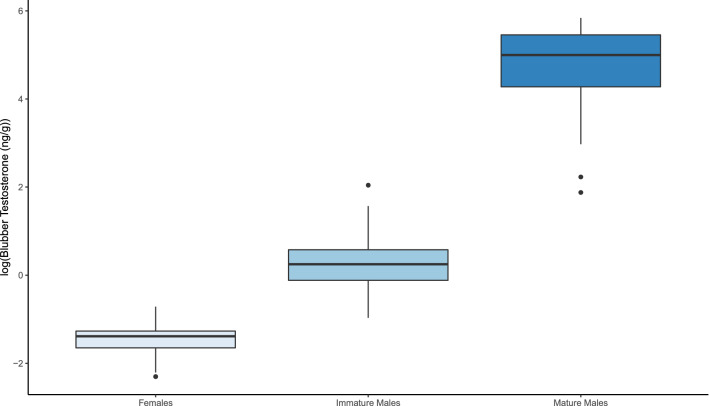
Mean blubber testosterone concentrations (ng/g lipid mass) for female, immature male, and mature male *T. truncatus* from Sarasota Bay, FL. Horizontal box lines represent the upper quartile, median, and lower quartile values. Whisker lines represent values within the 1.5 × interquartile range and the dots represent outliers.

Of the 55 individual animals sampled, 12 were females, 24 were immature males, and 19 were mature males based on records from the SDRP. A significant positive association was observed between log-transformed testosterone concentrations measured in the serum and those measured in the blubber across all paired male samples, excluding repeat samples of individuals (n = 29, r^2^ = 0.932, *p* < 2.2 × 10^–16^) (Fig. 1). This relationship was not found to be significant within maturity class (immature males: n = 14, r^2^ =  − 0.070, p = 0.705, mature males: n = 15, r^2^ = 5.39 × 10^–3^, *p* = 0.319). We found a significant difference between immature males and mature males in mean serum testosterone concentrations (n = 34, *p* = 1.344 × 10^–6^) with mature males (22.593 ± 3.017 SE ng/mL) having an average of more than 300 times the amount of testosterone than immature males (0.073 ± 0.009 SE ng/mL). A distinction was also seen in mean blubber testosterone concentrations among all three demographics (*p* = 3.02 × 10^–15^). Females (0.253 ± 0.032 SE ng/g) had an average of six times less blubber testosterone than immature males (1.568 ± 0.211 SE ng/g), which were almost a 100-fold lower than the average for mature males (147.671 ± 18.534 SE ng/g) (Fig. 2).

**Figure 3:**
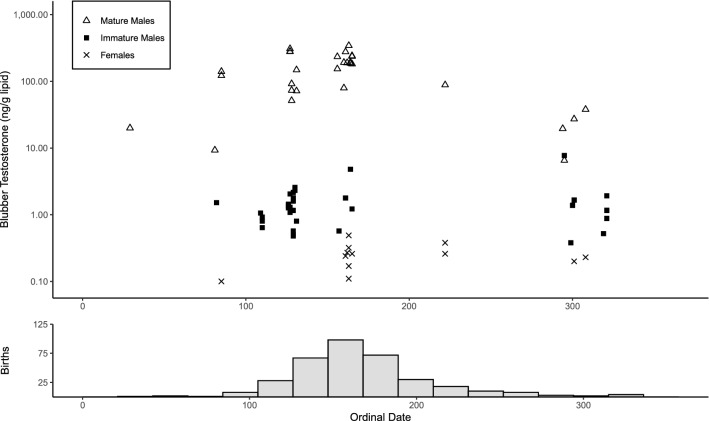
Top: Blubber testosterone (ng/g lipid mass) concentrations by ordinal date sampled for each demographic group of *T. truncatus* from Sarasota Bay, FL. Bottom: Unpublished data by R. S. Wells, 2020, frequency of *T. truncatus* birthdates from 1976–2020 (n = 353) binned in 3-week intervals in Sarasota Bay, FL. The expectation is that testosterone measurements would be the greatest before and near the peak calving period as gestation is estimated to be approximately 12.5 months, and testosterone increases are associated with seasonal testis development^54^.

Blubber testosterone concentrations showed significant variation by season only in mature males. No significant difference among seasons was found for females (*p* = 0.239) or immature males (*p* = 0.961). For mature males, mean testosterone levels in spring were greater than those in winter (*p* = 0.009) or fall (*p* = 5.55 × 10^–5^) (Fig. 3). Mean percent lipid concentrations were lower in blubber biopsies taken via dart biopsy compared to biopsies taken during health assessments though this difference was only significant in immature males (*p* = 0.009: also see Supplementary Data).

**Figure 4:**
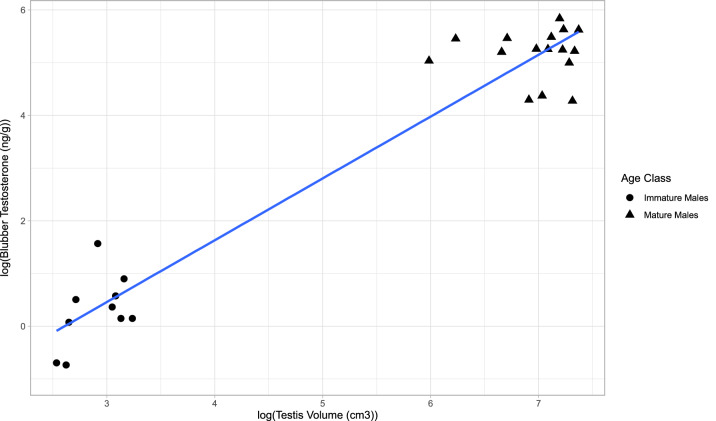
Relationship of blubber testosterone (ng/g lipid mass) concentration to estimated testis volume (cm^3^) for mature and immature male *T. truncatus* from Sarasota Bay, FL (r^2^ = 0.936, *p* = 7.977 × 10^–16^, y = 1.173*x + −3.060).

A significant positive linear relationship was observed between log-transformed blubber testosterone concentrations and log-transformed estimated testis volume across all males (n = 26, r^2^ = 0.936, *p* = 7.977 × 10^–16^) (Fig. 4). When we examined this correlation within each maturity class, we found no significant relationship in mature males (n = 16, r^2^ =  − 0.071, *p* = 0.975) or immature males (n = 10, r^2^ = 0.172, *p* = 0.129). Similarly, this positive linear relationship was seen between log-transformed serum testosterone concentrations and log-transformed estimated testis volume across all males (n = 26, r^2^ = 0.933, *p* = 7.977 × 10^–16^). Within maturity class, there was a slight relationship found among mature males (n = 16, r^2^ = 0.225, *p* = 0.042) but not immature males (n = 10, r^2^ = 0.034, *p* = 0.285) between serum testosterone and estimated testis volume. We found a significant difference between immature males and mature males in estimated testis volume (*p* = 2.519 × 10^–9^) with mature males (1144.9 ± 90.24 SE cm^3^) having an average of 60 times greater estimated testis volume than those of immature males (18.89 ± 1.48 SE cm^3^).

## Discussion

The periodic health assessments performed on the Sarasota Bay bottlenose dolphins provide a unique opportunity to carry out comparative studies on wild animals. We successfully quantified and analyzed testosterone concentrations from serum and blubber in males of known ages. A strong relationship between blubber and serum testosterone concentrations, such as the one found during this study, suggests that measuring a value using one matrix would allow one to infer similar information on maturity when using the other. This increases confidence in utilizing remote dart biopsy techniques for acquiring population-level data for free-swimming bottlenose dolphins in a minimally invasive manner. While other hormone values have been compared between these two matrices, this study is unique in that it compares testosterone directly between serum and blubber, with a greater sample size, and in a wild population. Due to the difficulty in obtaining serum from free-swimming cetaceans, such comparisons are uncommon but essential.

We found that testosterone levels were orders of magnitude higher in mature males than in immature males, both in serum and blubber, corroborating results of previous studies^1,6,23,30^. Unlike other studies however, we were also able to demonstrate that female bottlenose dolphins could be plausibly distinguished from males of any age based on blubber testosterone concentrations. Richard et al. (2017) found testosterone measurements in respiratory vapor, another remote sampling matrix being evaluated for applicability in endocrine assessments, lacked the strength to make this discernment in belugas^28^. This further strengthens the use of remote dart biopsy as a tool to uncover valuable demography data.

The significant relationship between serum and blubber testosterone should serve as a validation that concentrations in the blubber track levels in the serum. We see this correlation most strongly across the large difference between sexually immature and mature males. The dynamics of this relationship appear to be limited; however, as we see no correlation between serum and blubber within maturity class. Daily fluctuations in serum testosterone are not likely to be reflected in the blubber as it takes longer for steroid hormones to accumulate in and turn over in blubber relative to the blood^34,35^. Still, it is important to note that overall population trends persist despite the finer scale variability. Similarly, although we found blubber testosterone indicative of testis volume at scales that differentiate mature and immature animals this relationship is less substantial at finer scales such that predictions of the testis volume from blubber testosterone concentrations lack the precision to make useful estimates of volume within maturity classes.

The mean blubber testosterone concentration in mature males remained high and distinguishable from immature males across seasons. It is possible these findings are unique to common bottlenose dolphins, or delphinids in general, as large changes in seasonal testosterone values have been observed in mysticete whales, making it difficult to distinguish maturity in non-peak seasons^29^. Some of the outlier males in this study, particularly 10-year-old F264, were likely transitioning into sexual maturity during the sampling effort (see supplemental data); however, we could not untangle the seasonality patterns from these potential developmental ones. Nonetheless the presence of these potential pubertal males serves to strengthen the dataset.

While testosterone levels were still significantly higher than those found in immature males throughout the year, seasonality in blubber testosterone concentrations was, in fact, detected in mature males. We observed notably higher concentrations in mature males in the spring relative to the fall and winter months. This is consistent with observed patterns in serum testosterone^7^ and fits with the documented breeding season for bottlenose dolphins along the west coast of Florida^36^, including Sarasota Bay^37^. Extensive seasonal comparison was hindered by the lack of sampling year-round. Fifty-five of the 79 blubber samples were obtained in spring during health assessments performed by the SDRP, and no immature males were sampled at all during the summer. While we do not anticipate that it would change these results, year-round sampling and analysis should be performed to bolster them.

Adult male testosterone values appear to be stable through time within a given season. This was observed in two mature males of the same age that were each sampled in 2013 and again in 2019 (See F128 and F138 in supplemental material). Interestingly, F128 had about a third of the blubber testosterone concentration of F138 in 2013 and this difference was observed again after the 6 year sampling gap. Note that F128 was not in good health as a calf, and he is not as large as other males his age (7 cm less than asymptotic length for males)^38^. Both F128 and F138 are believed to have sired calves. This also highlights the individual variability in mature male testosterone production, even though the associated concentrations are still high enough to be distinguishable from immature male concentrations. Explanations for a lower average testosterone level include contaminant exposure, stress, and group social dynamics. Trego et al. (2018) showed a correlation between impaired testosterone production and high levels of halogenated organic compounds in the blubber of mature male common dolphins^39^. F128 had a summed polychlorinated biphenyl (PCB) concentration of 37.01 mg/kg (lipid normalized, unpublished data) in May 2013; F138 had about half that (19.30 mg/kg) at the same time. For perspective, Kannan et al. (2000) identified a threshold of total PCB concentration of 17 mg/kg as a threshold for the onset of reproductive impairment and immune suppression in marine mammals^40^. Jepson et al. (2005) identified a threshold of 17 mg/kg for total PCBs relative to adverse health effects in harbor porpoises (*Phocoena phocoena*)^41^.

We approximated the volume of the testes by assuming they are cylindrical. Although this method of estimation of testis size is the best that can be made with the available data, it overestimates the true volume of the imaged testes. However, from first principles this estimate should be approximately proportional to the true testis volume and therefore should provide an informative metric when examining the relationship between testosterone concentration and gonadal volume. It is typical of mammals to have testosterone levels commensurate with the size of their testes^42,43^. Previous studies on cetaceans have found testis mass to be strongly correlated with both serum^44^ and blubber^1^ testosterone concentrations. Therefore, the significant difference in average estimated testis size between immature males and mature males seen here was anticipated. The strong relationship found between blubber testosterone concentrations and estimated testis volume in this study serves to provide additional support for the use of blubber testosterone as a tool to differentiate immature from mature males in bottlenose dolphins.

The average percent lipid in the blubber samples was lower in those obtained via dart biopsy compared to those taken during capture-release health assessments. Generally, lower lipid values in dart biopsies are expected and are theorized to be a result of compression forces of the dart’s impact on the target animal’s blubber tissue^45^. Kellar et al. (2009) found that testosterone concentrations vary with percent lipid, leading us to correct for lipid in our blubber testosterone values^1^. The difference in percent lipid values between dart and health assessment biopsy was only significant in the immature males, which was unexpected, and warrants further investigation.

This study demonstrates the value of using minimally invasive dart biopsies to obtain population data commensurate with data obtained from serum samples collected during capture-release projects with common bottlenose dolphins. It further substantiates the ability to determine male maturity from blubber samples and incorporates the utility of distinguishing males from females. This would be useful in a population of unknown individuals to determine sex ratios and number of breeding males. Nevertheless, this must be validated for use in other species by obtaining baseline blubber testosterone values for known mature and immature males.

## Methods

### Samples

The SDRP collected 34 serum and 79 blubber samples during routine health assessments and dart sampling of live bottlenose dolphins in Sarasota Bay, Florida during 2011–2019. Small groups of selected dolphins were encircled with a 500 m × 4 m seine net deployed from a fast net boat in shallow waters^46^. Once securely and safely restrained by handlers, each dolphin was transferred to foam pads on the shaded deck of a 9-m-long, specially designed veterinary examination vessel. The dolphin was continuously monitored by veterinarians while full assessments and sampling were performed prior to being returned to the water and released. Blubber and blood samples were obtained and stored as described elsewhere^47,48^. Briefly, after application of anesthetic consisting of 2% lidocaine with epinephrine and rinsing of the site with chlorhexidine solution and methanol, a full-depth (epidermis to muscle), wedge-shaped blubber biopsy was surgically removed, typically from the dolphin's left side, approximately 10 cm ventral and 10 cm caudal to the posterior insertion of the dorsal fin. Blood was drawn from a prominent vessel in the tail fluke into blood collection tubes. Immediately following collection, blood was centrifuged to isolate the serum fraction, and blubber was sub-sampled using solvent-rinsed and autoclaved instruments. Both serum and blubber were placed in 2 ml polypropylene cryovials and frozen in a liquid nitrogen vapor shipper. After transport to the analytical laboratory, samples were stored at − 80 °C until analysis. Serum testosterone measurements were obtained via immunoassay using standard diagnostic laboratory procedures.

Twenty-eight blubber samples were also obtained via dart biopsy of free-swimming bottlenose dolphins in Sarasota Bay using methods described in Kellar et al.^49^. A 10 mm diameter stainless steel tip attached to a projectile dart was aimed at the animal’s lateral flank between the anterior insertion of the dorsal fin and the mid-point of the peduncle. Blubber/skin was sub-sampled using solvent-rinsed and autoclaved instruments. Blubber was placed in 2 ml polypropylene cryovials and frozen in a liquid nitrogen vapor shipper, before being stored frozen at  − 80 °C prior to processing.

### Ultrasonography

Evaluations were performed by an experienced marine mammal clinician using a portable ultrasound unit (SonoSite Edge, SonoSite, Bothell, WA) and a curvilinear 2–5 MHz transducer with a maximum depth of 30 cm. Testicles were examined either with the animal floating sternally in the water, using seawater for acoustic coupling, or with the animal in sternal or lateral recumbency on the deck of an examination vessel, using acoustic coupling gel. For the purpose of this study, only testicular diameter (or width) and estimated length were measured using the technique previously described^31^. Briefly, the maximum diameter of the testicle was determined by evaluating the entire organ, and measured in a transverse, cross-sectional plane. Testicles were examined in the transverse plane to identify the most cranial and caudal aspects of the testicle, which were marked on the skin surface of the lateral body wall. The distance measured between the two points was the estimated length.

### Ethical statement

Research was conducted under National Marine Fisheries Service Scientific Research Permits Nos. 522-1785, 15543, and 20455, and with Institutional Animal Care and Use Committee approvals renewed annually by Mote Marine Laboratory. All methods were performed in accordance with the relevant guidelines and regulations.

### Blubber hormone extraction

Hormone extraction followed the methods described by Kellar et al. (2015) with modifications^50^. Briefly, approximately 0.068–0.135 g of full-depth blubber was homogenized three times at a speed of 5 m/s for 45 s intervals in stainless steel microvials with a ¼” ceramic bead and 1000 μl of 100% ethanol. The contents of the homogenization tube were pipetted into a glass tube and the homogenization tube was rinsed twice with a total of 1400 µl of ethanol. The homogenate solution was combined with 2 mL of a 4:1 ethanol:acetone mixture before being vortexed for 5 min and then centrifuged for 15 min at 5000 RPM. The supernatant was transferred to a new glass tube and evaporated. 2 mL of diethyl ether was added to the evaporated contents, vortexed and centrifuged again. The supernatant was collected and evaporated. The glass tubes used in this step were weighed prior to and after evaporation to obtain the blubber lipid weight. This residue was then suspended in 1.5 mL of acetonitrile, vortexed, and 1.5 mL of hexane was added. After the solution was vortexed, centrifuged, and placed in a -20 ℃ freezer for at least an hour to promote separation, the acetonitrile layer was aspirated into a new glass tube. This process was repeated with another addition of 1.5 mL of hexane before the final acetonitrile solution was collected and evaporated. The remaining residue was centrifuged at 5000 RPM for 5 min before being suspended in 250 µl of 0.01 M phosphate buffered saline with bovine serum albumin and vortexed for 15 min. This solution was stored at -20 ℃ until used for the enzyme immunoassay. Proportion of extracted lipid was calculated by dividing the blubber lipid weight (g) by the total blubber weight (g) of each individual sample.

### Testosterone enzyme immunoassay

Assays were conducted following protocols described in Kellar et al. 2009. Blubber testosterone concentrations were determined using a commercially available enzyme immunoassay (EIA) kit by Enzo Life Sciences. The manufacturer-reported inter-assay coefficient of variation (COV) ranged from 3.4 to 7.0%, and intra-assay COV ranged from 4.1 to 5.0%, with a standard curve range between 0.1 and 25 ng/mL. The five highest documented cross-reactive steroids for the assay were as follows: testosterone at 100%, 5-dihydrotestosterone at 6.6%, 5-androstane-3,17-diol at 2.2%, 11-oxotestosterone at 1.8%, and androstenedione at 0.9%. It should be noted that the assay signal is a composite of these immunoreactive androgens; therefore, blubber testosterone concentrations reported here represent this aggregate of androgens (in nanograms per gram of blubber, wet weight) in which testosterone is very likely the most prevalent^1,17^.

### Extraction efficiency and controls

Extraction efficiency was determined for each set of extractions following the methods described in Kellar et al. 2009. Selected subsamples were spiked with six dilutions of testosterone ranging from 0 to 5 ng in the matrix tubes before initial homogenization. We extracted and quantified the testosterone in these subsamples according to the procedure described above. The resulting extraction efficiency rate was estimated as the percentage recovered in the final quantification after correcting for the intrinsic amount measured in the non-spiked samples^1^. The average efficiency rate across all extractions was 80.6% (range 63.4%–96.7%).

### Data analysis

Three demographics were categorized as follows: females, immature males (under 10 years old), and mature males (10 years old or older). Ten years of age is when summer serum testosterone concentrations have been reported to increase markedly in Sarasota males, along with increases in testis length and diameter^30^, and it falls within the range of ages believed to encompass when a male bottlenose dolphin becomes sexually mature^51,52^. Three males, not considered Sarasota Bay residents, were of unknown age and were labeled as immature as these were likely to be young, previously unobserved members of this population. One male was known to be “greater than 9 years old” and was designated as a mature male given the higher likelihood of being 10 or more years of age. Seasons were assigned as winter (January–March), spring (April–June), summer (July–September), and fall (October–December).

Testosterone concentrations (ng/ml for serum, ng/g for blubber) were natural log-transformed prior to analysis to reduce inherent heteroscedastic variation given the wide range of values among demographic groups. Serum values that were below the assay detection limit were used as half the assays lowest concentration standard value. Blubber testosterone concentration was corrected for the amount of lipid in the sample by dividing the ng/g mass by the percent lipid to get ng/g lipid mass. Testis volumes were estimated using maximum diameter and maximum length measurements taken from the left testis during health assessments. Testis volume was calculated by assuming cylindrical shape and using the formula:

$$ V_{testis} = \pi \left( {\frac{{D_{testis} }}{2}} \right)^{2} *L_{testis} $$

where V = volume, D = diameter, and L = length.

Linear regression analysis was used to determine the relationship between serum and blubber testosterone concentrations and between blubber testosterone concentrations and estimated testis volume in males. Single-factor ANOVAs and post hoc Tukey tests were used to compare blubber testosterone concentrations among each of the three demographic groups and among seasons for each demographic group. Welch two sample t-tests were performed to determine if the percent lipid differed by the way biopsies were obtained for each demographic. Results were considered significant when *p* < 0.05. Statistical analysis and figure generation was performed in *R* (version 3.6.3)^53^.

All data generated or analyzed during this study are included in this published article (and its Supplementary Information files).

## Supplementary Information

Supplementary Information.
