# Supplementary material for: Serum correlation, demographic differentiation, and seasonality of blubber testosterone in common bottlenose dolphins, Tursiops truncatus, in Sarasota Bay, FL
Source: Sci Rep. 2021 Apr 26;11:8941. doi: 10.1038/s41598-021-88602-z (PMC8076180; doi:10.1038/s41598-021-88602-z)
Supplement: Supplementary file 1 — Supplementary Information. [file 41598_2021_88602_MOESM1_ESM.pdf]

Serum correlation, demographic differentiation, and seasonality of blubber testosterone in common bottlenose dolphins, *Tursiops truncatus,* in Sarasota Bay, FL

Kathryn K. Sherman[1,*], Nicole E. Beaulieu-McCoy[2], Elyse L. Wurster[1], Randall S. Wells[3] , Cynthia R. Smith[4],
Aaron A. Barleycorn[3], Jason B. Allen[3], & Nicholas M. Kellar[5]

[1]Environmental Assessment Services, LLC., under contract to Southwest Fisheries Science Center, National Marine Fisheries Service, National Oceanic and Atmospheric Administration, 8901 La Jolla Shores Drive, La Jolla, CA 92037, USA

[2]Volunteer with Southwest Fisheries Science Center, National Marine Fisheries Service, National Oceanic and Atmospheric Administration, 8901 La Jolla Shores Drive, La Jolla, CA 92037, USA

[3]Chicago Zoological Society's Sarasota Dolphin Research Program, c/o Mote Marine Laboratory, 1600 Ken Thompson Parkway, Sarasota, FL 34236, USA

[4]National Marine Mammal Foundation, 2240 Shelter Island Drive, Suite 200, San Diego, CA 92106, USA

[5]Southwest Fisheries Science Center, National Marine Fisheries Service, National Oceanic and Atmospheric Administration, 8901 La Jolla Shores Drive, La Jolla, CA 92037, USA

*Kathryn.sherman@noaa.gov

SampleID	Biopsy via Dart	Sex	YR	MO	DA	Blubber Testosterone (ng/g mass)	% lipid	Blubber Testosterone (ng/g lipid)	Serum Testosterone (ng/ml)	Age at Sampling (yrs)	Total Length (cm)	Estimated Testis Volume (cm^3)
1318 (JA062)	1	F	2018	10	31	0.0	0.2	0.20	na	2	na	
1792 (JA061)	1	M	2018	10	29	0.1	0.3	0.38	na	3	na	
2271 (AB061)	1	M	2018	11	21	0.4	0.3	1.16	na	4	na	
B340	1	M	2016	4	20	0.3	0.3	0.92	na	na	na	
B341	1	M	2016	4	20	0.1	0.2	0.64	na	na	na	
B342	1	M	2016	4	20	0.3	0.3	0.80	na	na	na	
C250 (JA063)	1	M	2018	10	31	0.5	0.3	1.66	na	2	na	
C258 (AB055)	1	M	2018	10	30	0.2	0.1	1.38	na	7	na	
F123	0	F	2018	6	12	0.2	0.6	0.27	na	20	237	
F128	0	M	2019	6	10	13.4	0.2	79.36	13.2	27	257	1134.11
F128-T3	0	M	2013	5	8	25.7	0.4	73.40	19.1	21	257	1003.46
F128-T5	0	M	2013	5	8	22.8	0.2	92.01	na	21	257	
F128-T8	0	M	2013	5	8	15.6	0.3	51.39	13.94	21	257	
F138	0	M	2019	6	10	7.7	0.0	191.88	na	27	na	1196.57
F138-T3	0	M	2013	5	7	92.8	0.3	279.03	42.1	21	274	1383.62
F138-T5	0	M	2013	5	7	88.3	0.3	280.18	na	21	274	
F138-T8	0	M	2013	5	7	76.9	0.3	307.73	42.8	21	274	
F142 (JA069)	1	M	2019	1	29	2.8	0.1	19.96	na	27	na	
F146	0	M	2018	6	15	30.5	0.1	241.45	13.03	22	275	1234.01
F176 (JA071)	1	M	2019	3	25	33.6	0.2	140.46	na	26	na	
F178	0	M	2018	6	14	15.1	0.1	185.28	10.09	23	273	1532.12
F178 (FB178) (2016)	0	M	2016	5	11	52.3	0.4	148.34	45.64	21	272	1457.7
F188	0	M	2018	6	14	13.8	0.1	192.87	11.13	22	257	1075.21
F188 (FB188) (2016)	0	M	2016	5	11	31.2	0.4	71.96	19.09	20	257	1502.07
F196 (JA058)	1	M	2018	10	24	3.8	0.2	19.54	na	20	na	
F213	0	F	2018	6	11	0.1	0.5	0.24	na	11	235	
F228	0	M	2018	6	15	60.4	0.3	181.89	14.26	19	276	779.31
F238	0	M	2019	6	12	57.6	0.3	189.57	27.5	17	263	1370.01
F242 (JA064)	1	M	2018	11	8	5.6	0.1	37.83	na	28	na	
F246	0	M	2018	6	15	36.0	0.2	236.30	19.88	14	253	819.96
F250-T3	0	M	2013	5	9	0.4	0.3	1.16	<0.04	8	230	22.9
F250-T5	0	M	2013	5	9	0.8	0.4	1.78	na	8	230	
F250-T8	0	M	2013	5	9	0.7	0.3	2.16	na	8	230	
F252-T3	0	M	2013	5	6	0.4	0.3	1.44	0.04	7	238	21.11
F252-T5	0	M	2013	5	6	0.5	0.4	1.27	na	7	238	
F252-T8	0	M	2013	5	6	0.5	0.3	1.35	0.11	7	238	
F264	0	M	2019	6	6	15.6	0.1	153.75	4.97	11	239	397.61
F264 (JA059)	1	M	2018	10	25	1.4	0.2	7.72	na	10	na	
F264 (JA070)	1	M	2019	3	21	2.2	0.2	9.30	na	11	na	
F266	0	M	2019	6	6	73.3	0.3	233.94	23.8	12	244	509.15
F268 (JA072)	1	M	2019	3	25	35.3	0.3	122.07	na	26	na	
F270 (JA067)	1	M	2018	11	21	0.3	0.3	0.88	na	9	na	
F270-T3	0	M	2013	5	7	0.5	0.5	1.08	0.14	4	201	14.14
F270-T5	0	M	2013	5	7	0.7	0.6	1.29	na	4	201	
F270-T8	0	M	2013	5	7	0.6	0.3	2.05	0.17	4	201	
F275	0	F	2018	6	13	0.1	0.2	0.32	na	6	224	
F276	0	M	2018	6	11	32.2	0.1	277.08	33.65	26	281	1590.43
F277	0	F	2018	6	13	0.1	0.4	0.17	na	6	236	
F278 (JA068)	1	M	2018	11	21	0.5	0.3	1.92	na	8	na	
F280-T3	0	M	2013	5	9	0.8	0.5	1.66	0.06	3	214	15.08
F280-T5	0	M	2013	5	9	0.7	0.4	1.78	na	3	214	
F280-T8	0	M	2013	5	9	0.8	0.5	1.60	na	3	214	
F282-T3	0	M	2013	5	10	0.7	0.3	2.46	0.07	5	231	23.56
F282-T5	0	M	2013	5	10	0.8	0.4	2.31	na	5	231	
F282-T8	0	M	2013	5	10	0.9	0.4	2.57	0.1	5	231	
F283 (AB058)	1	F	2018	11	8	0.1	0.3	0.23	na	3	na	
F286	0	M	2018	6	14	0.8	0.2	4.80	<0.10	6	213	18.47
F286 (AB064)	1	M	2019	3	22	0.2	0.2	1.52	na	7	na	
F291 (AB065)	1	F	2019	3	25	0.0	0.4	0.10	na	2	na	
F292	0	M	2016	5	9	0.3	0.6	0.50	<0.10	4	216	12.61
F294	0	M	2016	5	11	0.4	0.5	0.80	<0.10	3	202	
F296	0	M	2017	5	8	0.5	0.4	1.16	<0.15	4	228	25.45
F296 (JA056)	1	M	2016	4	19	0.4	0.3	1.06	na	3	na	
F298	0	M	2017	5	9	0.2	0.3	0.57	<0.15	2	193	
F298 (JA066)	1	M	2018	11	21	0.4	0.3	1.17	na	3	na	
F306	0	M	2017	5	9	0.2	0.4	0.48	<0.15	4	208	13.78
F308	0	M	2018	6	11	0.4	0.2	1.78	<0.10	2	192	21.8
F310 (AB060)	1	M	2018	11	19	0.1	0.2	0.52	na	2	na	
F312	0	M	2018	6	15	0.3	0.3	1.22	<0.10	2	192	
F316	0	M	2019	6	7	0.2	0.3	0.57	<0.10	2	190	
F318	0	M	2019	6	13	66.1	0.2	343.67	29.9	>10	263	1333.26
F318 (AB057)	1	M	2018	10	31	5.8	0.2	27.23	na	>9	na	
FB54 (F54)	0	F	2018	6	13	0.1	0.5	0.11	na	47	248	
FB79 (F79)	0	F	2018	6	13	0.1	0.1	0.49	na	39	250	
FB90 (F90)	0	F	2018	6	15	0.0	0.2	0.26	na	48	262	
PHL2 (B346)	1	F	2016	8	12	0.1	0.2	0.38	na	>8	na	
SVRT (AB052)	1	F	2016	8	12	0.1	0.2	0.26	na	>1	na	
TMSP (JA056B)	1	M	2016	8	12	9.2	0.1	88.45	na	>25	na	
WLET (JA060)	1	M	2018	10	25	0.9	0.1	6.54	na	>12	na	
